# Supplementary material for: Application of Flow Cytometry Using Advanced Chromatin Analyses for Assessing Changes in Sperm Structure and DNA Integrity in a Porcine Model
Source: Int J Mol Sci. 2024 Feb 6;25(4):1953. doi: 10.3390/ijms25041953 (PMC10888687; doi:10.3390/ijms25041953)
Supplement: Supplementary file 1 [file ijms-25-01953-s001.zip › ijms-2814705-SI.pdf]

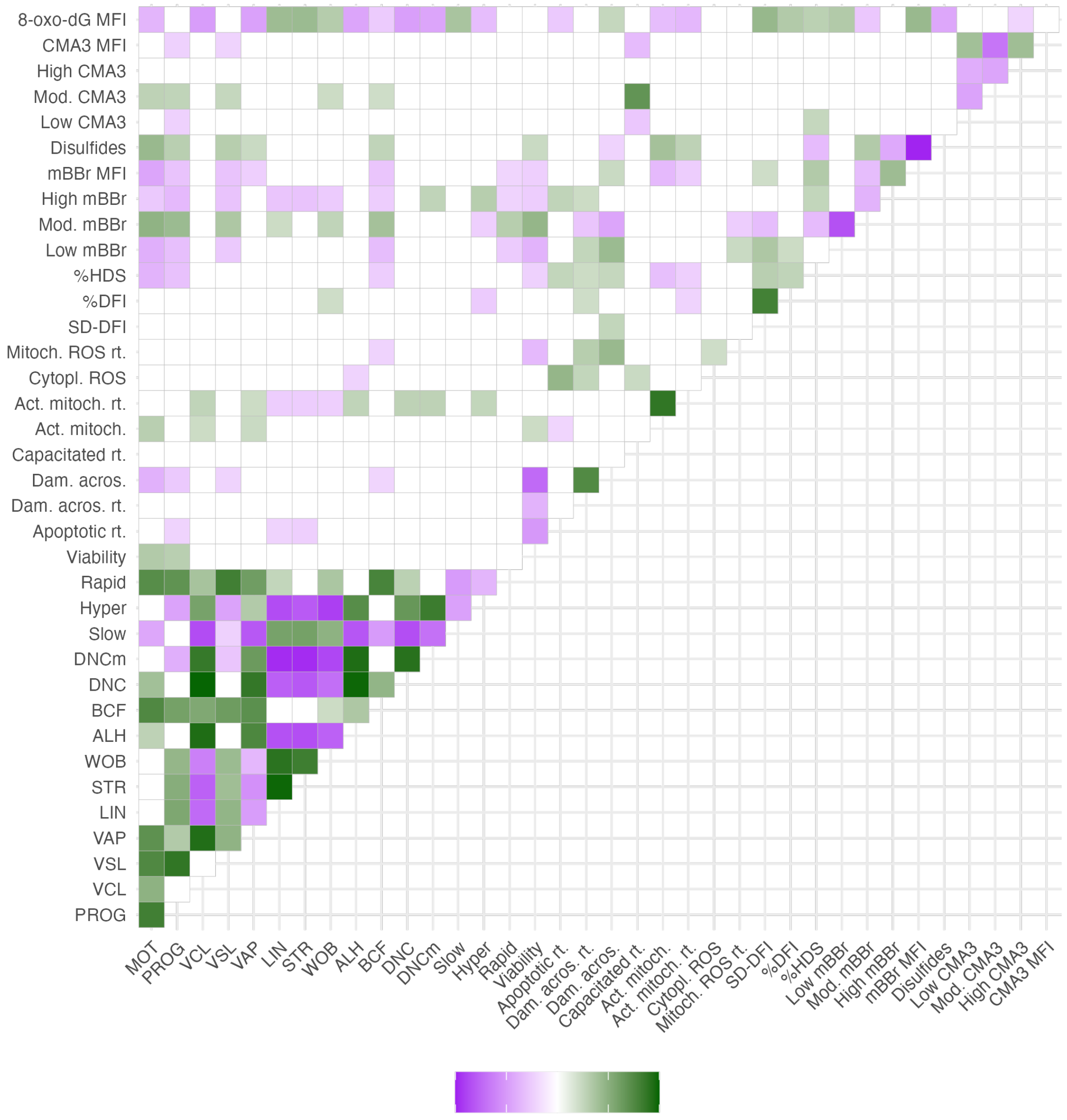

Figure S1. Correlation analysis for all the variables at day 0 of the experiment. The color gradient shows the r value. Correlations with P>0.05 (unadjusted) were removed (blank spaces). Variable names and standard abbreviations are explained in the text (rt.: ratio).

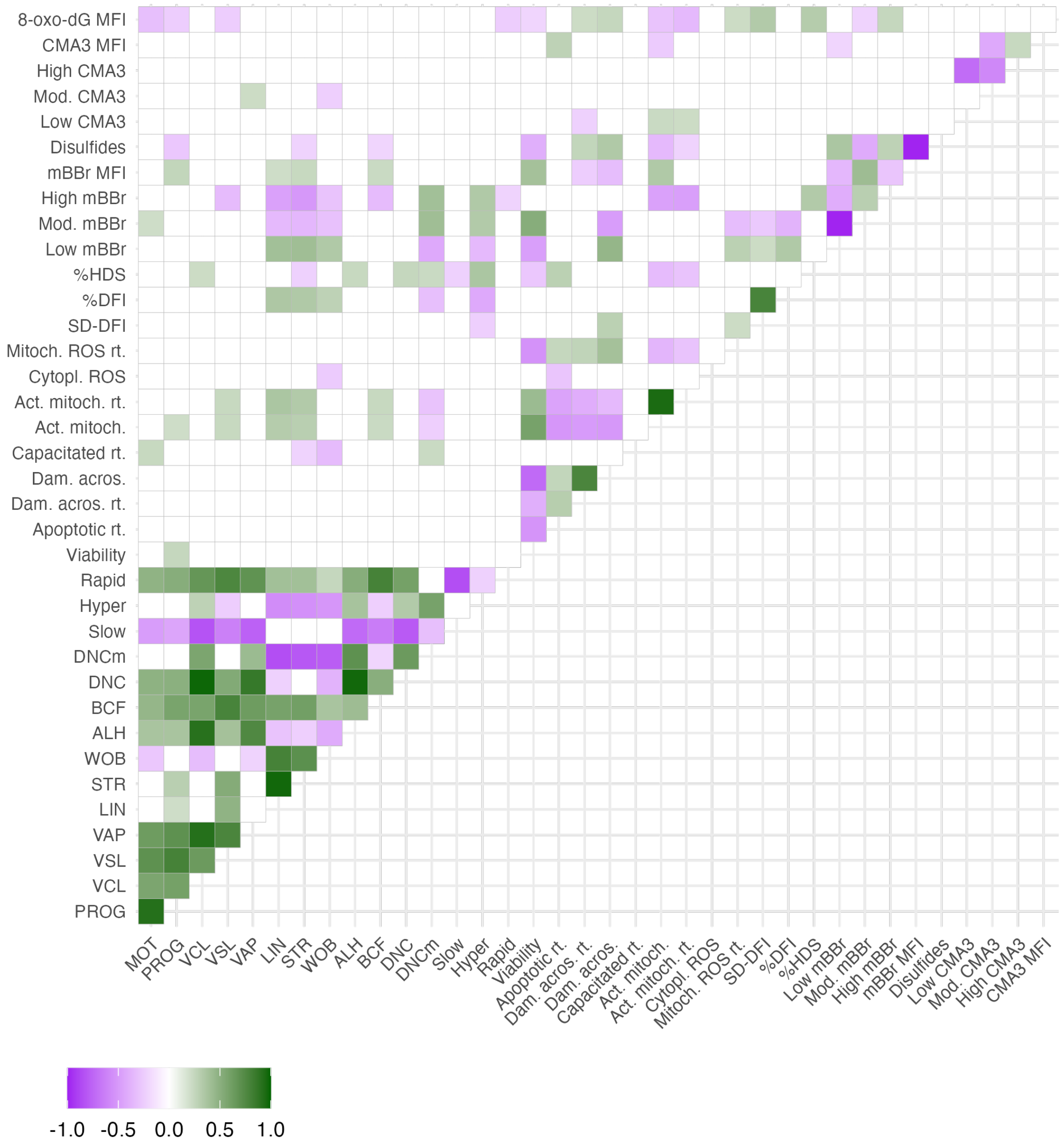

Figure S2. Correlation analysis for all the variables on day 11 of the experiment. The color gradient shows the r value. Correlations with P>0.05 (unadjusted) were removed (blank spaces). Variable names and standard abbreviations are explained in the text (rt.: ratio).

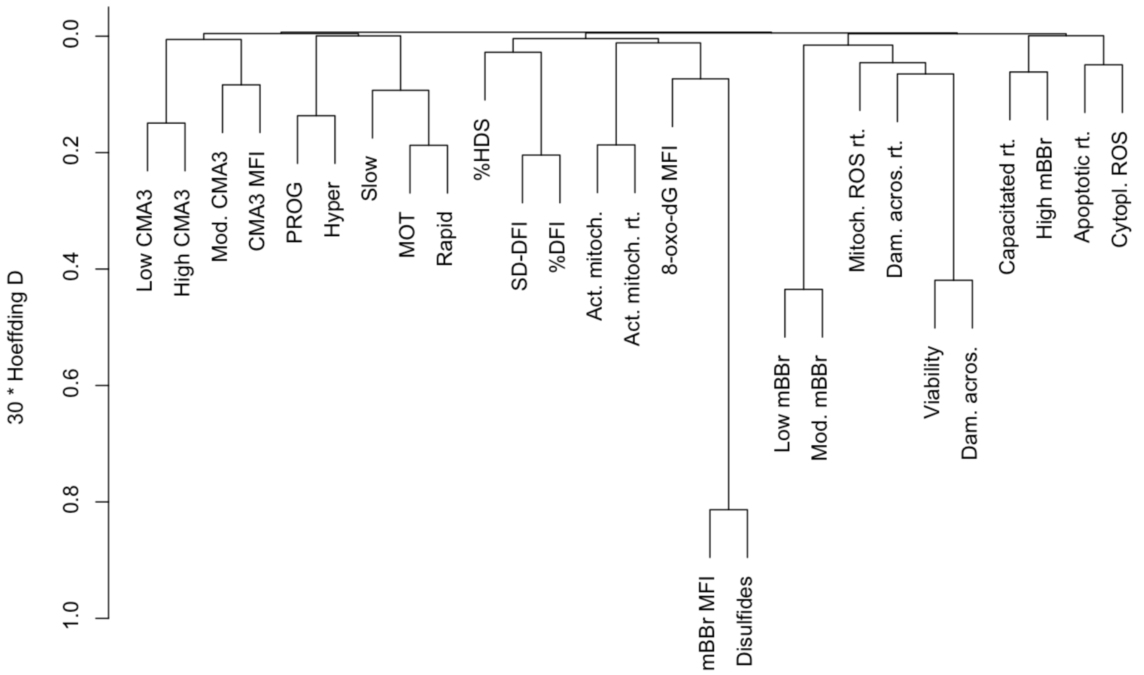

Figure S3. Hierarchical clustering of all the variables at day 0 of the experiment using the Hoeffding D statistic (times 30). Variables with shorter distances are more related and clustered together.

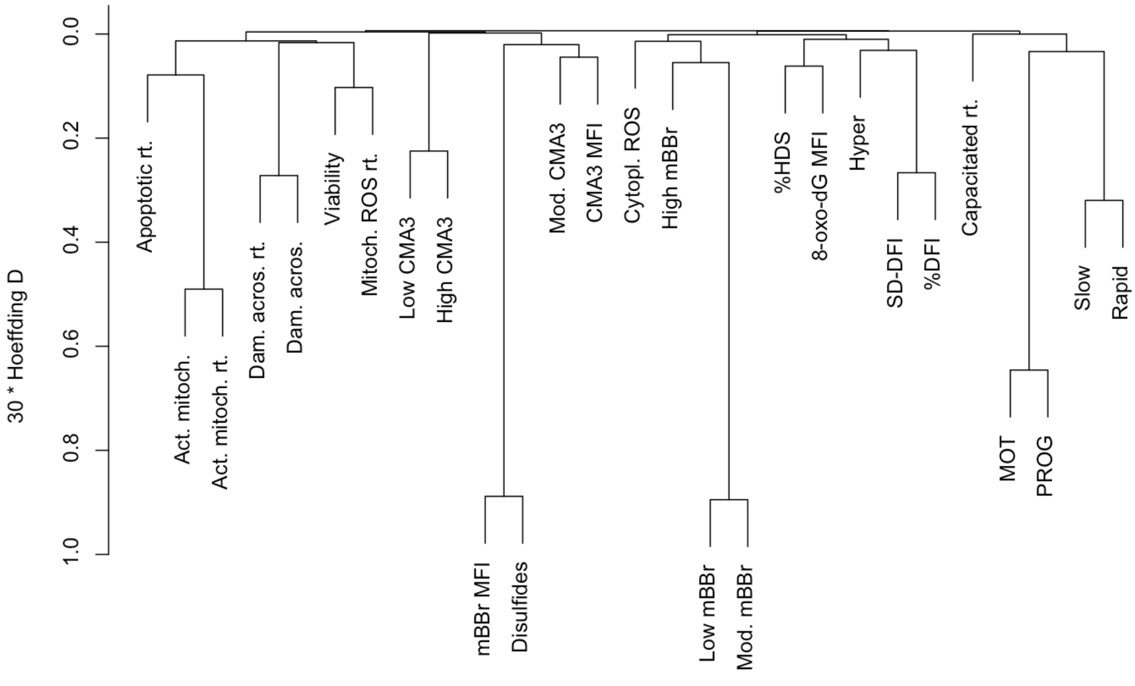

Figure S4. Hierarchical clustering of all the variables on day 11 of the experiment using the Hoeffding D statistic (times 30). Variables with shorter distances are more related and clustered together.

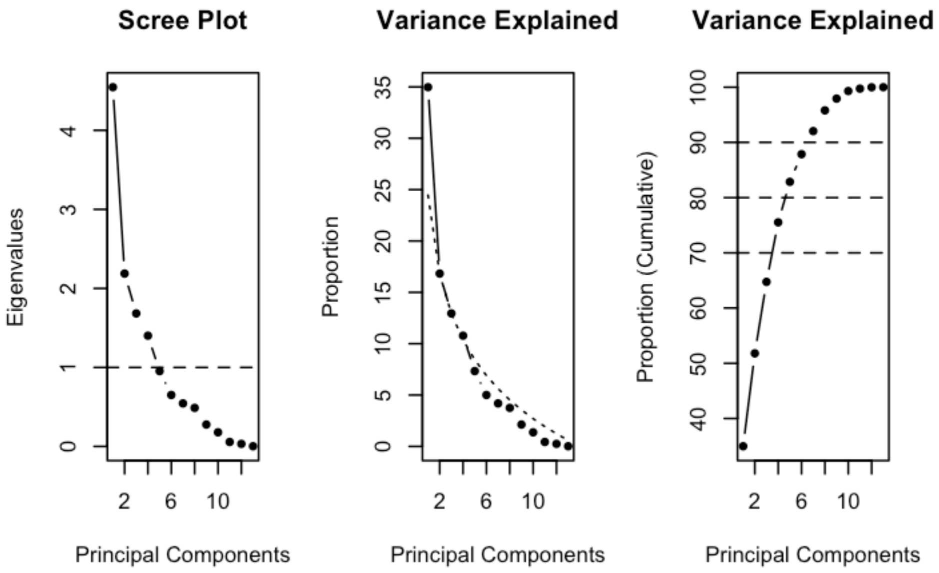

Figure S5. Scree plot for the PCA using the chromatin status variables (see Fig. 3 and Table 3) and proportion of the variance explained by the principal components (individual and cumulative). The Kaiser-Guttman criterion (eigenvalue >1, horizontal line in the scree plot) and the Explained Variance criterion (70% to 80% cumulative) suggest retaining four principal components. In the middle plot (variance explained for the individual variables), the dotted line indicates the expected variance explained; principal components are retained until the variance explained falls below this line.

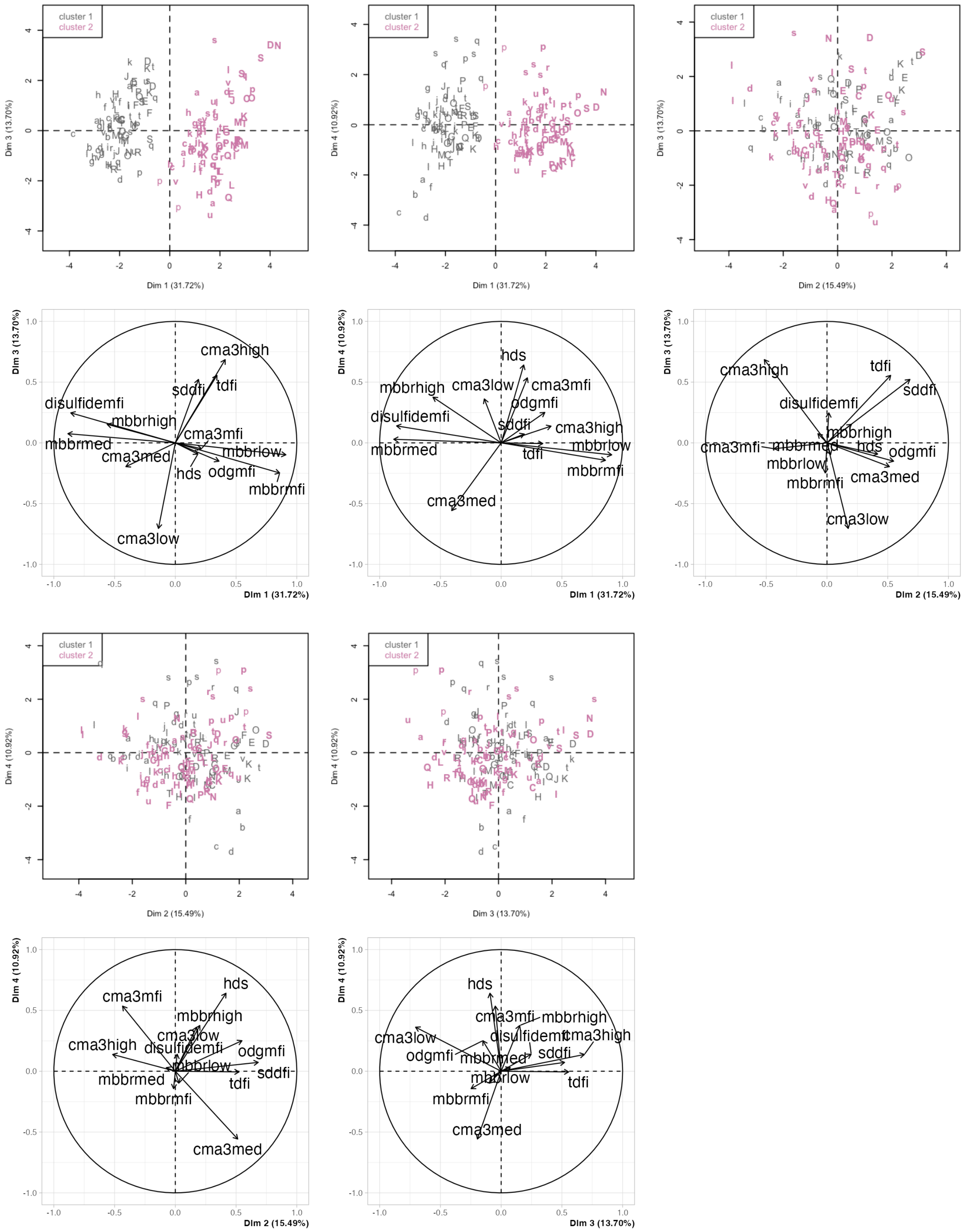

Figure S6. Principal component analysis (PCA) using the chromatin variables (PC1 vs. PC2 displayed in Fig. 3). In each row, the top panels display the observations in the space defined by the respective two principal components (PC, labeled as Dim), identified by boar (letters), day of analysis (regular font for D0 and bold for D11), and cluster (dark grey for cluster 1 and pink for cluster 2; hierarchical clustering on the PCA results). The bottom panel shows the variable loadings for the two first PCs (influence of the original

variables on the PC). Abbreviations are defined as: sddfi: SD-DFI; tdfi: %DFI; hds: %HDS; mbbrlow: Low mBBr; mbbrmed: Moderate mBBr; mbbrhigh: High mBBr; mbbrmfi: mBBr MFI; disulfidemfi: Disulfide levels; cma3low: Low CMA3; cma3med: Moderate CMA3; cma3high: High CMA3; cma3mfi: CMA3 MFI; odgmfi: 8-oxo-dG MFI.

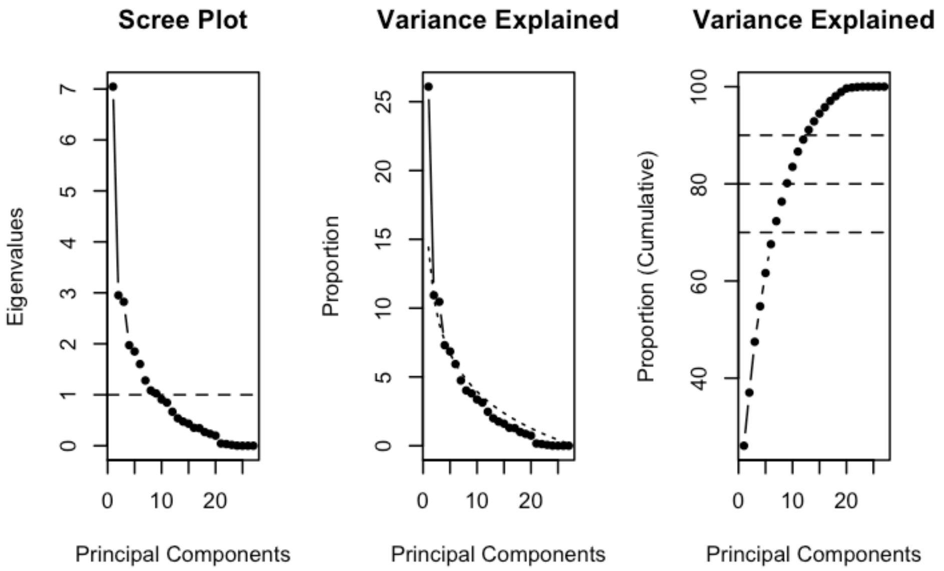

Figure S7. Scree plot for the PCA using the chromatin status variables (see Fig. 3 and Table 3) and proportion of the variance explained by the principal components (individual and cumulative). The Kaiser-Guttman criterion (eigenvalue >1, horizontal line in the scree plot) and the Explained Variance criterion (70% to 80% cumulative) suggest retaining eight principal components. In the middle plot (variance explained for the individual variables), the dotted line indicates the expected variance explained; principal components are retained until the variance explained falls below this line.

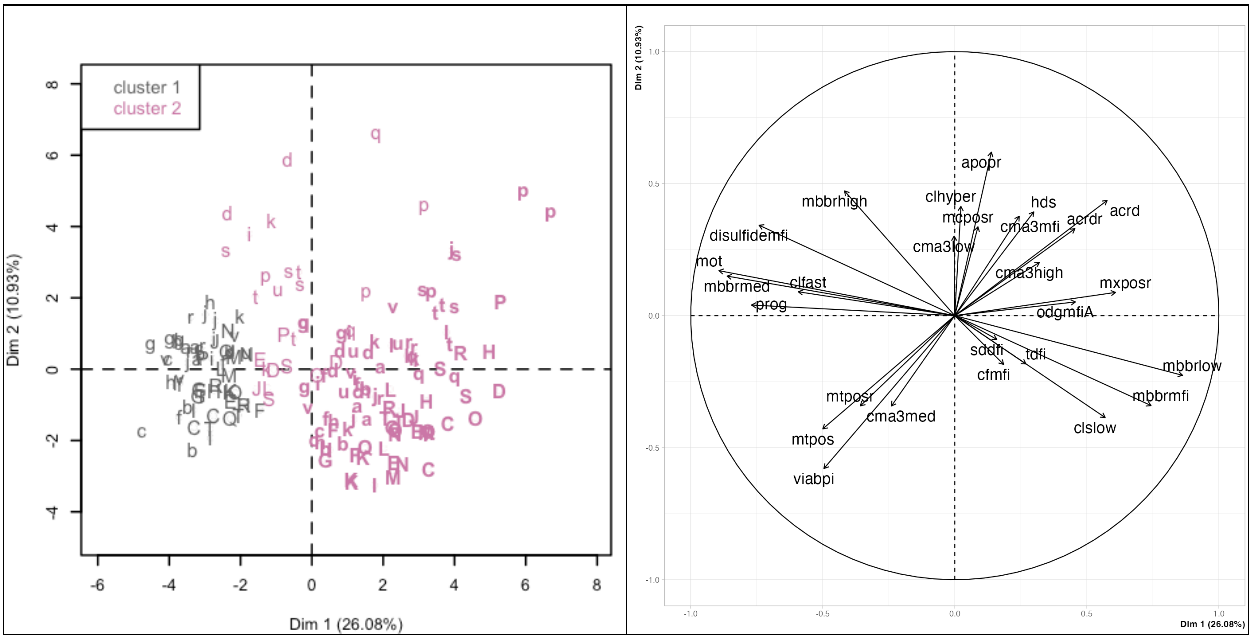

Figure S8. Principal component analysis (PCA) using all the variables in the study. The left panel shows the observations in the space defined by the first two principal components (PC, labeled as Dim 1 and Dim 2), identified by boar (letters), day of analysis (plain for day 0 and bold for day 11), and cluster (dark grey for cluster 1 and pink for cluster 2). The right panel shows the variable loadings for the two first PCs (influence of the original variables on the PC).

mot: Total motility; prog: Progressive motility; clslow: Slow subpopulation; clhyper: Hyperactivated-like subpopulation; clfast: Fast subpopulation; viabpi: Viable (PI^{-}); apopr: Apoptotic (ratio); acrdr: Acrosomal damage (ratio); acrd: Acrosomal damage (total); mcposr: Capacitated ($M540^{+}$, ratio); mtpos: Active mitochondria; mtposr: Active mitochondria (ratio); cfmfi: Cytoplasmic ROS (MFI); mxposr: Mitochondrial ROS (ratio); sddfi: SD-DFI; tdfi: %DFI; hds: %HDS; mbbrlow: Low mBBr; mbbrmed: Moderate mBBr; mbbrhigh: High mBBr; mbbrmfi: mBBr MFI; disulfidemfi: Disulfide levels; cma3low: Low CMA3; cma3med: Moderate CMA3; cma3high: High CMA3; cma3mfi: CMA3 MFI; odgmfiA: 8oxodG MFI.

Table S1. Mean ± SEM for the sperm motility and physiology (flow cytometry) parameters for each analysis day and P values for the factor effects (LME analysis with day, boar, and their interaction).

Variable	Day 0	Day 11	Day	Boar	Day×Boar
Total motility (%)	75.51±1.63	25.73±1.62	<0.001	<0.001	<0.001
Progressive motility (%)	38.23±1.52	13.68±1.48	<0.001	<0.001	<0.001
VCL (µm/s)	96.14±2.25	73.37±2.23	<0.001	<0.001	0.118
VSL (µm/s)	23.78±0.60	19.04±0.58	<0.001	<0.001	0.005
VAP (µm/s)	53.73±1.04	37.93±1.02	<0.001	<0.001	0.136
LIN (%)	30.73±1.00	30.47±0.99	0.466	<0.001	0.055
STR (%)	53.21±1.18	57.18±1.16	0.061	<0.001	0.090
WOB (%)	58.02±0.68	54.90±0.67	<0.001	<0.001	0.092
ALH (µm)	2.29±0.05	2.05±0.05	0.091	<0.001	0.055
BCF (Hz)	15.68±0.38	10.76±0.37	<0.001	<0.001	0.023
DNC (µm²/s)	241.42±10.63	157.97±10.53	<0.001	<0.001	0.068
DNCm (µm)	10.45±0.53	7.60±0.52	0.561	<0.001	0.129
Slow (%)	30.66±1.49	48.20±1.46	<0.001	<0.001	0.024
Fast (%)	49.94±1.59	34.43±1.55	<0.001	<0.001	0.009
Hyperactivated (%)	19.40±1.22	17.37±1.22	0.098	<0.001	0.060
Viability (%)	84.02±0.66	80.36±0.65	<0.001	<0.001	0.002
Apoptotic, ratio (%)	1.82±0.16	1.84±0.16	0.003	<0.001	0.217
Damaged acrosomes, ratio (%)	3.17±0.50	6.36±0.49	0.003	0.193	0.655
Damaged acrosomes (%)	14.36±0.67	19.35±0.66	<0.001	<0.001	0.869
Capacitated, ratio (%)	0.76±0.10	1.07±0.09	0.009	0.704	0.946
Active mitochondria (%)	78.28±1.71	67.46±1.69	<0.001	<0.001	<0.001
Active mitochondria, ratio (%)	89.37±1.98	81.55±1.96	<0.001	<0.001	<0.001
Cytoplasmic ROS (MFI)	5.94±0.74	8.16±0.70	0.102	0.482	0.999
Mitochondrial ROS, ratio (%)	3.49±0.39	7.91±0.39	<0.001	<0.001	0.006

VCL, curvilinear velocity; VSL, straight-line velocity; VAP, average path velocity; LIN, linearity of the curvilinear trajectory (ratio of VSL/VCL); STR, straightness; ALH, amplitude of lateral head displacement; WOB, wobble coefficient; BCF, beat cross-frequency; DNC, sperm dance; DNCm, sperm mean dance.

Table S2. Type III Analysis of Variance Table with Satterthwaite's method from the analysis of the motility and sperm physiology variables.

Total motility

	Sum Sq.	Mean Sq.	Num. d.f.	Den. d.f.	F value	Pr(<F)
**Day**	21724.66268	21724.66268	1	107.20	771.11	<0.001
**Boar**	6865.57649	196.15933	35	107.10	6.96	<0.001
**Day×Boar**	3105.62874	88.73225	35	107.20	3.15	<0.001

Progressive motility

	Sum Sq.	Mean Sq.	Num. d.f.	Den. d.f.	F value	Pr(<F)
**Day**	95.07067	95.07067	1	107.24	242.90	<0.001
**Boar**	69.99155	1.99976	35	107.12	5.11	<0.001
**Day×Boar**	38.65270	1.10436	35	107.24	2.82	<0.001

VCL

	Sum Sq.	Mean Sq.	Num. d.f.	Den. d.f.	F value	Pr(<F)
**Day**	0.15294	0.15294	1	107.44	36.13	<0.001
**Boar**	0.71265	0.02036	35	107.19	4.81	<0.001
**Day×Boar**	0.20151	0.00576	35	107.44	1.36	0.118

VSL

	Sum Sq.	Mean Sq.	Num. d.f.	Den. d.f.	F value	Pr(<F)
**Day**	58.57535	58.57535	1	107.31	46.72	<0.001
**Boar**	247.65126	7.07575	35	107.09	5.64	<0.001
**Day×Boar**	84.67760	2.41936	35	107.31	1.93	0.005

VAP

	Sum Sq.	Mean Sq.	Num. d.f.	Den. d.f.	F value	Pr(<F)
**Day**	0.30957	0.30957	1	107.26	109.70	<0.001
**Boar**	0.56329	0.01609	35	107.20	5.70	<0.001
**Day×Boar**	0.13118	0.00375	35	107.26	1.33	0.136

LIN

	Sum Sq.	Mean Sq.	Num. d.f.	Den. d.f.	F value	Pr(<F)
**Day**	0.05939	0.05939	1	107.61	0.54	0.466
**Boar**	27.56539	0.78758	35	107.34	7.11	<0.001
**Day×Boar**	5.87445	0.16784	35	107.61	1.51	0.055

STR

	Sum Sq.	Mean Sq.	Num. d.f.	Den. d.f.	F value	Pr(<F)
**Day**	21228.09813	21228.09813	1	109.00	3.57	0.061
**Boar**	1471018.31710	42029.09477	35	109.00	7.07	<0.001
**Day×Boar**	294275.66274	8407.87608	35	109.00	1.42	0.090

WOB

	Sum Sq.	Mean Sq.	Num. d.f.	Den. d.f.	F value	Pr(<F)
**Day**	0.00003	0.00003	1	107.43	13.73	<0.001
**Boar**	0.00055	0.00002	35	107.23	6.21	<0.001
**Day×Boar**	0.00013	0.00000	35	107.43	1.41	0.092

ALH

	Sum Sq.	Mean Sq.	Num. d.f.	Den. d.f.	F value	Pr(<F)
**Day**	0.05884	0.05884	1	109.00	2.92	0.091
**Boar**	2.99391	0.08554	35	109.00	4.24	<0.001
**Day×Boar**	1.06928	0.03055	35	109.00	1.51	0.055

BCF

	Sum Sq.	Mean Sq.	Num. d.f.	Den. d.f.	F value	Pr(<F)
**Day**	69.81130	69.81130	1	109.00	86.96	<0.001
**Boar**	119.40348	3.41153	35	109.00	4.25	<0.001
**Day×Boar**	47.18470	1.34813	35	109.00	1.68	0.023

DNC

	Sum Sq.	Mean Sq.	Num. d.f.	Den. d.f.	F value	Pr(<F)
**Day**	0.12467	0.12467	1	107.59	15.05	<0.001
**Boar**	1.31753	0.03764	35	107.32	4.54	<0.001
**Day×Boar**	0.42677	0.01219	35	107.59	1.47	0.068

DNCm

	Sum Sq.	Mean Sq.	Num. d.f.	Den. d.f.	F value	Pr(<F)
**Day**	0.02026	0.02026	1	107.62	0.34	0.561
**Boar**	12.73486	0.36385	35	107.35	6.11	<0.001
**Day×Boar**	2.79202	0.07977	35	107.62	1.34	0.129

Slow

	Sum Sq.	Mean Sq.	Num. d.f.	Den. d.f.	F value	Pr(<F)
**Day**	268.40612	268.40612	1	109.00	77.52	<0.001
**Boar**	559.22535	15.97787	35	109.00	4.61	<0.001
**Day×Boar**	201.90437	5.76870	35	109.00	1.67	0.024

Fast

	Sum Sq.	Mean Sq.	Num. d.f.	Den. d.f.	F value	Pr(<F)
**Day**	1417.25341	1417.25341	1	109.00	62.43	<0.001
**Boar**	3693.94122	105.54118	35	109.00	4.65	<0.001
**Day×Boar**	1462.76011	41.79315	35	109.00	1.84	0.009

Hyperactivated

	Sum Sq.	Mean Sq.	Num. d.f.	Den. d.f.	F value	Pr(<F)
**Day**	9.85061	9.85061	1	107.34	2.78	0.098
**Boar**	411.72853	11.76367	35	107.24	3.32	<0.001
**Day×Boar**	185.61660	5.30333	35	107.34	1.50	0.060

Viability

	Sum Sq.	Mean Sq.	Num. d.f.	Den. d.f.	F value	Pr(<F)
**Day**	5453470.89571	5453470.89571	1	107.23	31.16	<0.001
**Boar**	50758072.03956	1450230.62970	35	107.12	8.29	<0.001
**Day×Boar**	12626053.92236	360744.39778	35	107.23	2.06	0.002

Apoptotic (ratio)

	Sum Sq.	Mean Sq.	Num. d.f.	Den. d.f.	F value	Pr(<F)
**Day**	3.07432	3.07432	1	109.00	9.40	0.003
**Boar**	39.06730	1.11621	35	109.00	3.41	<0.001
**Day×Boar**	13.97482	0.39928	35	109.00	1.22	0.217

Damaged acrosomes (ratio)

	Sum Sq.	Mean Sq.	Num. d.f.	Den. d.f.	F value	Pr(<F)
**Day**	5.10383	5.10383	1	106.12	9.36	0.003
**Boar**	23.85832	0.68167	35	105.75	1.25	0.193
**Day×Boar**	16.84606	0.48132	35	106.12	0.88	0.655

Damaged acrosomes

	Sum Sq.	Mean Sq.	Num. d.f.	Den. d.f.	F value	Pr(<F)
**Day**	5.53396	5.53396	1	106.79	24.66	<0.001
**Boar**	28.56622	0.81618	35	106.79	3.64	<0.001
**Day×Boar**	5.62702	0.16077	35	106.79	0.72	0.869

Capacitated (ratio)

	Sum Sq.	Mean Sq.	Num. d.f.	Den. d.f.	F value	Pr(<F)
**Day**	687.59179	687.59179	1	108.00	6.98	0.009
**Boar**	2928.57239	83.67350	35	108.00	0.85	0.704
**Day×Boar**	2141.70697	61.19163	35	108.00	0.62	0.946

Active mitochondria

	Sum Sq.	Mean Sq.	Num. d.f.	Den. d.f.	F value	Pr(<F)
**Day**	21079516.13664	21079516.13664	1	106.54	48.18	<0.001
**Boar**	114757354.57737	3278781.55935	35	106.08	7.49	<0.001
**Day×Boar**	49435213.33291	1412434.66665	35	106.54	3.23	<0.001

Active mitochondria (ratio)

	Sum Sq.	Mean Sq.	Num. d.f.	Den. d.f.	F value	Pr(<F)
**Day**	11518785.46115	11518785.46115	1	109.00	16.07	<0.001
**Boar**	133070655.50010	3802018.72857	35	109.00	5.30	<0.001
**Day×Boar**	96457218.10832	2755920.51738	35	109.00	3.84	<0.001

Median of CM-H_2DCFDA fluorescence (cytoplasmic ROS)

	Sum Sq.	Mean Sq.	Num. d.f.	Den. d.f.	F value	Pr(<F)
**Day**	0.00901	0.00901	1	106.92	2.72	0.102
**Boar**	0.11587	0.00331	35	106.55	1.00	0.482
**Day×Boar**	0.04553	0.00130	35	106.92	0.39	0.999

Mitochondrial ROS (ratio)

	Sum Sq.	Mean Sq.	Num. d.f.	Den. d.f.	F value	Pr(<F)
**Day**	14.33921	14.33921	1	107.20	129.68	<0.001
**Boar**	11.73570	0.33531	35	107.10	3.03	<0.001
**Day×Boar**	7.35844	0.21024	35	107.20	1.90	0.006

Table S3. Type III Analysis of Variance Table with Satterthwaite's method from the analysis of the sperm chromatin status variables.

SD-DFI

	Sum Sq.	Mean Sq.	Num. d.f.	Den. d.f.	F value	Pr(<F)
**Day**	0.00000	0.00000	1	109.00	0.00	0.997
**Boar**	0.02764	0.00079	35	109.00	4.15	<0.001
**Day×Boar**	0.00662	0.00019	35	109.00	0.99	0.490

%DFI

	Sum Sq.	Mean Sq.	Num. d.f.	Den. d.f.	F value	Pr(<F)
**Day**	2.77196	2.77196	1	106.80	9.33	0.003
**Boar**	41.36496	1.18186	35	106.73	3.98	<0.001
**Day×Boar**	10.49102	0.29974	35	106.80	1.01	0.468

%HDS

	Sum Sq.	Mean Sq.	Num. d.f.	Den. d.f.	F value	Pr(<F)
**Day**	0.15137	0.15137	1	109.00	2.30	0.132
**Boar**	12.73631	0.36389	35	109.00	5.53	<0.001
**Day×Boar**	1.65134	0.04718	35	109.00	0.72	0.869

Low mBBr fluor.

	Sum Sq.	Mean Sq.	Num. d.f.	Den. d.f.	F value	Pr(<F)
**Day**	321.06583	321.06583	1	109.00	1940.57	<0.001
**Boar**	49.88082	1.42517	35	109.00	8.61	<0.001
**Day×Boar**	27.81707	0.79477	35	109.00	4.80	<0.001

Moderate mBBr fluor.

	Sum Sq.	Mean Sq.	Num. d.f.	Den. d.f.	F value	Pr(<F)
**Day**	148946833.94327	148946833.94327	1	109.00	1185.85	<0.001
**Boar**	23805819.55299	680166.27294	35	109.00	5.42	<0.001
**Day×Boar**	14144236.32929	404121.03798	35	109.00	3.22	<0.001

High mBBr fluor.

	Sum Sq.	Mean Sq.	Num. d.f.	Den. d.f.	F value	Pr(<F)
**Day**	66.30380	66.30380	1	107.17	673.85	<0.001
**Boar**	22.42663	0.64076	35	106.80	6.51	<0.001
**Day×Boar**	6.68840	0.19110	35	107.17	1.94	0.005

Median mBBr fluor.

	Sum Sq.	Mean Sq.	Num. d.f.	Den. d.f.	F value	Pr(<F)
**Day**	1.83180	1.83180	1	107.42	1210.43	<0.001
**Boar**	0.39361	0.01125	35	107.23	7.43	<0.001
**Day×Boar**	0.15850	0.00453	35	107.42	2.99	<0.001

Disulfide levels, fluor. median

	Sum Sq.	Mean Sq.	Num. d.f.	Den. d.f.	F value	Pr(<F)
**Day**	4595001.72693	4595001.72693	1	107.39	1273.00	<0.001
**Boar**	938206.51839	26805.90053	35	107.23	7.43	<0.001
**Day×Boar**	393602.37077	11245.78202	35	107.39	3.12	<0.001

Low CMA3 fluor.

	Sum Sq.	Mean Sq.	Num. d.f.	Den. d.f.	F value	Pr(<F)
**Day**	0.00209	0.00209	1	109.00	0.62	0.431
**Boar**	0.10437	0.00298	35	109.00	0.89	0.643
**Day×Boar**	0.12533	0.00358	35	109.00	1.07	0.385

Moderate CMA3 fluor.

	Sum Sq.	Mean Sq.	Num. d.f.	Den. d.f.	F value	Pr(<F)
**Day**	224973.46298	224973.46298	1	106.69	4.33	0.040
**Boar**	1584304.36958	45265.83913	35	106.43	0.87	0.674
**Day×Boar**	1990994.15434	56885.54727	35	106.69	1.09	0.355

High CMA3 fluor.

	Sum Sq.	Mean Sq.	Num. d.f.	Den. d.f.	F value	Pr(<F)
**Day**	0.02515	0.02515	1	106.95	19.23	<0.001
**Boar**	0.05213	0.00149	35	106.85	1.14	0.301
**Day×Boar**	0.04803	0.00137	35	106.95	1.05	0.412

Median CMA3 fluor.

	Sum Sq.	Mean Sq.	Num. d.f.	Den. d.f.	F value	Pr(<F)
**Day**	0.01125	0.01125	1	106.94	10.43	0.002
**Boar**	0.03740	0.00107	35	106.99	0.99	0.495
**Day×Boar**	0.02613	0.00075	35	106.94	0.69	0.893

8-oxo-dG MFI A

	Sum Sq.	Mean Sq.	Num. d.f.	Den. d.f.	F value	Pr(<F)
**Day**	0.01914	0.01914	1	107.63	15.41	<0.001
**Boar**	0.90970	0.02599	35	107.36	20.93	<0.001
**Day×Boar**	0.13873	0.00396	35	107.63	3.19	<0.001

Table S4. Loadings and contributions of each starting variable on the two principal components computed from the PCA. The loadings correspond to the correlations of variables and components. The contributions represent the importance of each variable for each component.

Variable	Loadings		Contributions	
	PC1	PC2	PC1	PC2
Total motility	-0.893	0.170	11.33	0.98
Progressive motility	-0.770	0.040	8.42	0.05
Slow	0.570	-0.386	4.61	5.05
Fast	0.023	0.414	0.01	5.80
Hyperactivated	-0.593	0.090	4.99	0.28
Viability	-0.496	-0.579	3.49	11.36
Apoptotic (ratio)	0.139	0.619	0.27	12.99
Damaged acrosomes (ratio)	0.455	0.329	2.94	3.67
Damaged acrosomes	0.577	0.436	4.73	6.45
Capacitated (ratio)	0.088	0.337	0.11	3.84
Active mitochondria	-0.500	-0.429	3.55	6.23
Active mitochondria (ratio)	-0.358	-0.342	1.82	3.96
Cytoplasmic ROS	0.185	-0.185	0.49	1.15
Mitochondrial ROS (ratio)	0.609	0.088	5.26	0.26
SD-DFI	0.159	-0.090	0.36	0.27
%DFI	0.270	-0.183	1.03	1.13
%HDS	0.299	0.393	1.27	5.23
Low mBBr fluor.	0.863	-0.226	10.57	1.73
Moderate mBBr fluor.	-0.862	0.150	10.55	0.76
High mBBr fluor.	-0.418	0.472	2.48	7.56
mBBr MFI	0.743	-0.341	7.85	3.93
Disulfide levels	-0.741	0.342	7.80	3.97
Low CMA3 fluor.	-0.003	0.301	0.00	3.07
Moderate CMA3 fluor.	-0.240	-0.342	0.82	3.97
High CMA3 fluor.	0.321	0.202	1.46	1.38
CMA3 MFI	0.244	0.376	0.85	4.80
8-oxo-dG MFI	0.457	0.052	2.96	0.09

VCL, curvilinear velocity; VSL, straight-line velocity; VAP, average path velocity; LIN, linearity of the curvilinear trajectory (ratio of VSL/VCL); STR, straightness; ALH, amplitude of lateral head displacement; WOB, wobble coefficient; BCF, beat cross-frequency; DNC, sperm dance; DNCm, sperm mean dance; SD-DFI, the standard deviation of the DFI (DNA fragmentation index); %DFI, DNA fragmentation; HDS, chromatin immaturity; mBBr, monobromobimane; CMA3, chromomycin A; MFI: Median fluorescent intensity for the fluorescent label.
